# PAD-MAC: Primary User Activity-Aware Distributed MAC for Multi-Channel Cognitive Radio Networks

**DOI:** 10.3390/s150407658

**Published:** 2015-03-30

**Authors:** Amjad Ali, Md. Jalil Piran, Hansoo Kim, Jihyeok Yun, Doug Young Suh

**Affiliations:** Department of Electronics and Radio Engineering, College of Electronics and Information, Kyung Hee University, Deogyeong-daero, Giheung-gu, Yongin-si, Gyeonggi-do 446-701, Suwon, Korea; E-Mails: amjad.ali@khu.ac.kr (A.A.); piran.mj@gmail.com (M.J.P.); puplepie@naver.com (H.K.); ychwh555@naver.com (J.Y.)

**Keywords:** primary user channel usage, traffic pattern analysis, multi-channel MAC, heterogeneous cognitive radio networks, channel selection, idle length estimation, predictive and fast channel switching, PHY-layer monitoring

## Abstract

Cognitive radio (CR) has emerged as a promising technology to solve problems related to spectrum scarcity and provides a ubiquitous wireless access environment. CR-enabled secondary users (SUs) exploit spectrum white spaces opportunistically and immediately vacate the acquired licensed channels as primary users (PUs) arrive. Accessing the licensed channels without the prior knowledge of PU traffic patterns causes severe throughput degradation due to excessive channel switching and PU-to-SU collisions. Therefore, it is significantly important to design a PU activity-aware medium access control (MAC) protocol for cognitive radio networks (CRNs). In this paper, we first propose a licensed channel usage pattern identification scheme, based on a two-state Markov model, and then estimate the future idle slots using previous observations of the channels. Furthermore, based on these past observations, we compute the rank of each available licensed channel that gives SU transmission success assessment during the estimated idle slot. Secondly, we propose a PU activity-aware distributed MAC (PAD-MAC) protocol for heterogeneous multi-channel CRNs that selects the best channel for each SU to enhance its throughput. PAD-MAC controls SU activities by allowing them to exploit the licensed channels only for the duration of estimated idle slots and enables predictive and fast channel switching. To evaluate the performance of the proposed PAD-MAC, we compare it with the distributed QoS-aware MAC (QC-MAC) and listen-before-talk MAC schemes. Extensive numerical results show the significant improvements of the PAD-MAC in terms of the SU throughput, SU channel switching rate and PU-to-SU collision rate.

## 1. Introduction

In the recent years, wireless communications and their applications have gained exponential growth, which has raised the demand for radio spectrum resources. Due to static spectrum allocation policies, the radio spectrum is running out; moreover, statistics show that its licensees are unable to fully utilize the radio spectrum and that between 15% and 80% of the assigned spectrum currently is being wasted in the most parts of the world [1]. A study conducted in the major cities of the United States in 2002 showed that the radio spectrum below 1 GHz remains mostly unused for long periods of time [2]. Therefore, the Federal Communication Commission (FCC) in the U.S. has begun to consider more flexible uses of the radio spectrum. The aim of the Next Generation program of the Defense Advanced Research Project Agency (DARPA) is to redistribute the allocated radio spectrum dynamically. Cognitive radio (CR) was introduced as the key technology for enabling dynamic spectrum access (DSA) [3] with the aim of providing a ubiquitous wireless access environment with seamless wireless connection [4,5]. CR-enabled secondary users (SUs) sense their surroundings in order to detect unused portions of the available radio frequencies and to adapt some of their operating parameters [6–9] accordingly.

**Figure 1:**
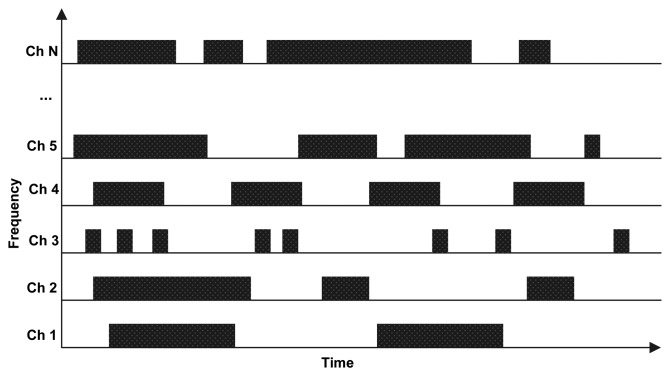
Primary user (PU) behavior on licensed channels.

Primary users (PUs) transmit a variety of applications with different traffic characteristics [10–12]. Hence, PU channel usage patterns over each licensed channel can vary and depend on the type of PU application; such behavior is illustrated in Figure 1. CR should be more than just a radio that takes immediate advantage of spectrum opportunities; it should also have the ability to learn from past experiences. Learning makes CR operations more efficient as compared to the case where the CR has only the information available when it was designed. Ideally, information collected during the lifetime of the CR should be used. However, the majority of research into cognitive radio networks (CRNs) has focused on methods that only use instantaneous information about the environment as a basis for dynamic operation. The available licensed channels for selection can be assumed to be equally good [13–15] or can be characterized based on their bandwidth [16–18] or interference level [19,20] to preferentially select the channels with the widest bandwidths or lowest interference levels. SUs sense their environments and react opportunistically to the estimated changes in the spectrum availability. Such a channel selection approach can result in the selection of a bad channel, because the SU randomly selects channels that could be heavily used by PUs (if that channel happened to be available during the channel sensing time). This may cause frequent service disruptions for SUs, since they have to refrain from transmission, resulting in severe interference to PUs and excessive channel switching, which results in low network throughput [21]. In addition, every instance of channel switching causes a non-negligible delay in the transmissions of SUs.

When multiple SUs come together to form single-hop networks, due to hardware variations in radio transceivers and spatial variations in PU licensed channel usage, different SUs in the networks can perceive different subsets of channels available to them for communication [22]. This heterogeneity in the available channel sets (*i.e.*, spectrum heterogeneity) across the network makes the network heterogeneous. Therefore, we refer to such CR-enabled networks as heterogeneous CRNs. In this paper, we investigate a PU activity-aware distributed medium access control (PAD-MAC) protocol for multi-channel heterogeneous CRNs with the objective of fully utilizing the advanced capabilities provided by CR to optimize network performance and avoid harmful interference with PUs. To achieve this goal, each SU monitors a list of licensed channels and estimates the idle slots over each monitored channel. PAD-MAC allocates the available channels to each individual SU based on cross-layer sensing, such as PHY-layer sensing used to monitor the PU traffic usage patterns and channel availability at any particular time. MAC-layer sensing is used to further avoid PU-to-SU or SU-to-SU interference. Moreover, PAD-MAC restricts each SU to exploiting the licensed channel only for the duration of the estimated idle time. The key contributions and results of this paper are as follows:

- We propose a PHY-layer-based channel usage pattern identification scheme using a two-state Markov model. We estimate the minimum transmission opportunity length (Min TOL) and maximum transmission opportunity length (Max TOL) using continuous PHY-layer channel monitoring. Furthermore, based on PHY-layer monitored parameters, we compute the rank of each individual idle channel, which quantify the SU transmission success or failure over the corresponding channel.
- By exploiting the knowledge of the estimated idle period, we introduce a predictive and fast channel switching scheme that maximizes SU spectrum occupancy and minimizes the disruption rate for PUs.
- We develop an imperative association among the Min TOL, Max TOL and standard deviation of the Min TOL that helps to predict the PU traffic patterns, future idle and busy slots for a particular licensed channel.
- We propose a channel-locking mechanism that reserves a channel for the transmission of SU and eliminates SU-to-SU interference.
- We design a PU activity-aware distributed MAC protocol for multi-channel heterogeneous CRNs with the prime objectives to maximize network capacity and to minimize the PU-to-SU collision rate and SU channel switching rate. PAD-MAC achieves these goals by selecting the optimal channel, utilizing it only for the duration of its estimated Min TOL and employing the fast and predictive channel switching scheme. Moreover, PAD-MAC also resolves SU-to-SU interference. We validate the performance of this protocol with the distributed QoS-aware MAC (QC-MAC) and listen-before-talk MAC to prove its significance.

The rest of the paper is organized as follows. In Section 2, we discuss some background knowledge and state-of-the-art research concerning channel selection and SU data transmission strategies. Section 3 outlines the system models. Section 4 presents the detailed overview and design objectives of the proposed PAD-MAC protocol for SU channel selection and data transmission. Section 5 describes the numerical results and experimental setups. In Section 6, we discuss the simulation results. Finally, the paper is concluded with some suggested future work in Section 7.

## 2. Related Work

In this section, we discuss some background information and state-of-the-art research in the area of MAC protocols for channel selection and data transmission for SUs. The design of MAC protocols for multi-channel CRNs differs from that of classic MAC protocols for wireless and wired networks. MAC protocols for CRNs should be closely coupled between physical and MAC layers, *i.e.*, spectrum opportunities that are detected on the physical layer and MAC layer perform spectrum sensing and allocation.

A decentralized cognitive MAC (DC-MAC) protocol for CRNs is proposed in [23], in which the authors introduced a new channel access policy that requires partial knowledge of the states of the channels for every SU. The DC-MAC uses the theory of the partially observable Markov decision process (POMDP), where the traffic characteristics of PUs are modeled using the Markov chain. This accesses channels on the basis of the expected reward-based suboptimal greedy approach. However, the implementation of DC-MAC is limited by the assumption that the transition probabilities in the Markov channel model are given, which is not yet feasible in practice. For efficient spectrum management in CRNs, a hardware-constrained MAC (HC-MAC) protocol [24] was proposed by Jia *et al.* to deal with channel access without requiring global synchronization among all SUs. A sender and receiver are required to become synchronized before data transmission and synchronization is performed using S-RTS /S-CTS messages. HC-MAC divides each time frame into three phases: the contention, sensing and transmission phases. A distributed multi-channel MAC for multi-hop CR (MMAC-CR) was proposed in [25]. MMAC-CR performs two stages of sensing: (1) fine sensing; and (2) cooperative detection. Channels are modeled using two states: Markov-chain ON-OFF states. If the sensed channel is determined to be idle, the SU sends a beacon packet in the corresponding mini slot via physical implementation of the AND rule. Channels are selected to minimize the expected interference, and the time frames are divided into two windows: (1) the *ad hoc* traffic indication message window; and (2) the DATA window. However, MMAC-CR requires tight synchronization within the network that strongly depends on the quality of the control channel. Moreover, use of the AND rule would be too aggressive with respect to the PUs.

Clustering-based MAC protocols for CRNs were proposed in [26,27]. In [26], Hamdaoui *et al.* introduced an opportunistic spectrum MAC (OS-MAC), based on coordination-based direct access in which SUs who want to communicate with each other are grouped together to form a cluster. Cluster heads are delegated SU nodes, which are responsible for acquiring the traffic load information of a channel and for propagating this information within their respective clusters. OS-MAC uses a probabilistic channel selection scheme to reduce the inter-cluster interference, and each time frame is divided into the select phase, update phase and delegate phase. However, OS-MAC does not address channel sensing and mobility functionalities; therefore, interference caused by PUs, which is a key role of CR, has not been implemented in OS-MAC. An opportunistic clustering-based spectrum access protocol for mesh CRNs was proposed in [27]. The objective of this proposal was to equally distribute the loads of SUs over the available licensed channels and to minimize the total amount of interference that is generated. The authors solved this problem using an integer linear programming (ILP)-based optimization. Each mesh router (MR), which also acts as a cluster head, shares the network state information, *i.e.*, the number of SUs, the positions of their APs, occupied primary channels and estimated interference generated by SU mesh nodes. Resource optimization for wireless mesh networks using MAC-layer channel reassignment method is introduced in [28]. Authors solve this problem using ILP and proposed a polynomial time heuristic algorithm.

Some other famous MAC protocols for CRNs are presented in [12,29–32]. Zhao *et al.* proposed a heterogeneous distributed MAC (HD-MAC) [12] that selects the channels, based on the traffic load, connectivity and interference. In HD-MAC, SUs are organized into local groups where the MAC structure is divided into the beacon period, coordination window and data transmission period. A distributed QoS-aware MAC (QC-MAC) protocol for multi-channel CRNs was proposed in [29]. This exploits the PU traffic patterns to determine the channels set for MAC-layer sensing and data transmissions. It specifically deals with QoS-aware transmissions with the objective of minimizing the PU-to-SU collision rate. An IEEE 802.11-based MAC protocol for mesh CRNs was proposed in [30]. Here, every SU is equipped with two CR transceivers; one is dedicated to control information, and the second is utilized for the data transmissions of SUs. Each SU node senses the available channels and selects the long-term residency (LR) channels for exchanging control information. The prime objective of this protocol is to limit the use of the control channel and reduce the possibility of saturation. However, finding network-wide LR channels is not a practical approach for opportunistic access networks. A group-based cooperative MAC (GC-MAC) protocol for enhancing the sensing accuracy in CRNs was proposed in [31]. An SU-selective technique was adopted for reducing the sensing overhead. The proposed protocol was well analyzed under saturation and non-saturation with time-invariant and time-varying channel conditions. Similarly, to jointly improve the spectrum sensing efficiency through cooperative sensing and system lifetime, an optimal scheduling scheme for sensor-aided CRN was studied in [32]. Sensors are divided into multiple non-disjointed subsets based on their individual channel conditions, and each subset is activated successively while all of the remaining sensors are kept in a low-energy sleep mode.

A distributed, direct access-based MAC protocol for CRNs (CMAC) was proposed in [33]. Here, each SU is equipped with a single half-duplex transceiver to detect the PUs in its vicinity and shares its sensing information with other neighboring SUs. CMAC divides the time frame into two parts: (1) the beacon period; and (2) data transfer period. Each SU periodically visits a common channel to obtain PU and SU discovery information for resynchronization. In [34], Liu *et al.* proposed a scalable hybrid MAC protocol for heterogeneous machine-to-machine networks. Their proposed protocol achieves hierarchical performance by using different contending priorities and incorporates both the persistent CSMSand TDMA schemes. Moreover, an incremental contention priority scheme was used to guarantee fair access among multiple heterogeneous devices. Cooperative sensing can improve the reliability of spectrum sensing. However, if the SUs are selfish, they may not take part in spectrum sensing. Therefore, an efficient MAC protocol may force selfish SUs to take part in cooperative sensing in order to improve the network performance. In this way, a fair, social welfare-correlated equilibrium technique was introduced in [35] to ensure the fairness of all SUs and to maximize the system utility.

Therefore, it would be beneficial to design a MAC protocol for CRNs that is fully aware of the activity of PUs and their traffic usage patterns over each licensed channel. This should provide some efficient way to enhance the SUs' throughput, minimize the PU-to-SU collision rate and minimize the SU channel switching rate. It should also provide an efficient/fast channel switching scheme to control the activity of SUs over licensed channels and to avoid harmful interference with SUs.

## 3. Preliminaries

### 3.1. System Model

To make the rest of this paper easy to follow, we list some frequently-used symbol notations and symbols terminologies in Table 1.

A group of SUs is assumed to form a single-hop CRN where each SU directly communicates with other SUs in its transmission range. In the MAC protocol design, an important concern is sender and receiver synchronization due to the spectrum heterogeneity and the contention for multiple SUs for the available spectrum. However, licensed channels may not always be available to SUs; therefore, we use the common control channel (CCC) for exchanging control information and contentions among SUs. There is a tradeoff between PHY-layer spectrum sensing/monitoring and cost. However, PHY-layer sensing is significantly important due to the heterogeneous PUs, whose traffic usage patterns depend on the applications type of the PUs [11,29]. Therefore, each SU is equipped with two radios, which is similar to approach employed by Jiang *et al.* [36]. One radio is dedicated to continuous PHY-layer monitoring, while the second is reserved for data transmissions.

**Figure 2:**
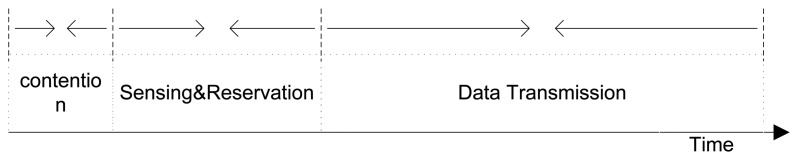
Secondary user (SU) frame architecture.

The CRN consists of a total of N licensed channels that can be used for the transmission of SUs. Each estimated idle slot for any licensed channel is divided into three segments: (1) contention; (2) MAC-layer sensing and channel reservation; and (3) SU data transmission; this concept is illustrated in Figure 2. Avoiding SU-to-SU interference is another prime concern in MAC protocol design for CRNs. However, the IEEE.802.22 standard for CRNs [37] suggests that it should be dealt with in the context of internetwork coordination of spectrum sensing and allocation. In our proposed PAD-MAC, we deal with SU-to-SU interference during MAC-layer sensing and channel reservation via a channel-locking mechanism; this will be discussed in detail in Section 3.5.

### 3.2. Estimation of Min TOL and Max TOL Using PHY-Layer Channel Monitoring

In heterogeneous CRNs, every channel follows a unique usage pattern, which can vary over time and is generally dependent on the types of PU applications [11,29]. Transmission opportunities length (TOL) is the key part of enabling opportunistic-access CRNs. Therefore, PU activity-aware estimation of TOLs plays an important role in enhancing the throughput of CRNs and controlling the activity of SUs within the licensed bands [38]. With the help of estimated TOLs, SUs can efficiently exploit the licensed channels without causing harmful interference with the PUs and can make intelligent and fast handoff decisions. In this section, we present the procedure of proposed PHY-layer channel monitoring scheme, and then, based on the continuous PU activity monitoring, we present a procedure (*i.e.*, Algorithm 1) to estimate the Min TOL and Max TOL. The PHY-layer channel monitoring scheme collects the licensed channel usage patterns and observes the PU behavior for a sufficient period of time (*i.e.*, sampling time). Further, based on the reasonable past observations, we can predict that if a particular channel is idle, it may remain idle at least for the duration of its estimated Min TOL.



**Algorithm 1:** Expected Min TOL and Max TOL.
 **Input:**
${\text{FreeTime}}_{t}^{i}$, 
$\mathrm{Min}\;\text{Idle}\;{\text{Length}}_{t}^{i}$, 
$\mathrm{Max}\;\text{Idle}\;{\text{Length}}_{t}^{i}$ and *Identifier* **Output:**
$\mathrm{Min}\;{\text{TOL}}_{t}^{i}$
*and*
$\mathrm{Max}\;{\text{TOL}}_{t}^{i}$ **begin**  
$\mathrm{Min}\;{\text{TOL}}_{t}^{i}\leftarrow 0$  
$\mathrm{Max}\;{\text{TOL}}_{t}^{i}\leftarrow 0$   **if**
*Identifier* == *TRUE*
**then**    
$\mathrm{Min}\;\text{Idle}\;{\text{Length}}_{t}^{i}\leftarrow \text{Free}\;{\text{Time}}_{t}^{i}$    
$\mathrm{Max}\;\text{Idle}\;{\text{Length}}_{t}^{i}\leftarrow \text{Free}\;{\text{Time}}_{t}^{i}$  **else**   **if**
$\text{Free}\;{\text{Time}}_{t}^{i}\leq \mathrm{Min}\;Idel\;{\text{Length}}_{t-1}^{i}$
**then**     
$\mathrm{Min}\;\text{Idle}\;{\text{Length}}_{t}^{i}\leftarrow \frac{\mathrm{Min}\;\text{Idle}\;{\text{Length}}_{t-1}^{i}+\text{Free}\;{\text{Time}}_{t}^{i}}{2}$   **if**
$\text{Free}\;{\text{Time}}_{t}^{i}\geq \mathrm{Max}\;Idel\;{\text{Length}}_{t-1}^{i}$
**then**     
$\mathrm{Min}\;\text{Idle}\;{\text{Length}}_{t}^{i}\leftarrow \frac{\mathrm{Min}\;\text{Idle}\;{\text{Length}}_{t-1}^{i}+\text{Free}\;{\text{Time}}_{t}^{i}}{2}$     
$\mathrm{Max}\;\text{Idle}\;{\text{Length}}_{t}^{i}\leftarrow \frac{\mathrm{Max}\;\text{Idle}\;{\text{Length}}_{t-1}^{i}+\text{Free}\;{\text{Time}}_{t}^{i}}{2}$   **if**
$\text{Free}\;{\text{Time}}_{t}^{i}>\mathrm{Min}\;\text{Idle}\;{\text{Length}}_{t-1}^{i}\&\&\text{Free}\;{\text{Time}}_{t}^{i}<\mathrm{Max}\;\text{Idle}\;{\text{Length}}_{t-1}^{i}$
**then**    
$\mathrm{Min}\;\text{Idle}\;{\text{Length}}_{t}^{i}\leftarrow \frac{\mathrm{Min}\;\text{Idle}\;{\text{Length}}_{t-1}^{i}+\text{Free}\;{\text{Time}}_{t}^{i}}{2}$   
$\mathrm{Min}\;{\text{TOL}}_{t}^{i}\leftarrow \mathrm{Min}\;\text{Idle}\;{\text{Length}}_{t}^{i}$   
$\mathrm{Max}\;{\text{TOL}}_{t}^{i}\leftarrow \mathrm{Min}\;\text{Idle}\;{\text{Length}}_{t}^{i}$


Algorithm 1: The identifier value is provided TRUEonly for the first time at *t* = *t*_1_. Therefore, at *t*_1_, $\mathrm{Min}\;{\text{TOL}}_{t}^{i}$ and $\mathrm{Max}\;{\text{TOL}}_{t}^{i}$ are the same as the observed $\text{Free}\;{\text{Time}}_{t}^{i}$. However, for the following times, we take the average of the monitored $\text{Free}\;{\text{Time}}_{t}^{i}$ and previously-computed idle length $\mathrm{Min}\;\text{Idle}\;{\text{Length}}_{t-1}^{i}$ or $\mathrm{Max}\;\text{Idle}\;{\text{Length}}_{t-1}^{i}$ according to the situation to reflect the respective change of PU activity in the estimation of idle slots.

**Figure 3:**
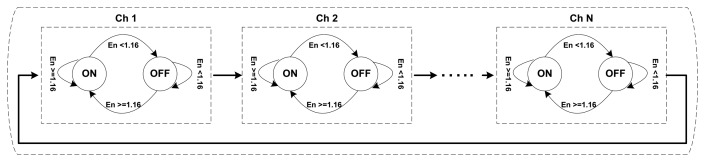
PU activity monitoring architecture.

We model each channel as an ON-OFF source alternating between OFF (idle) and ON (busy) periods where PU arrivals and utilization are modeled using an exponential distribution, which is similar to [39-41]. The PHY-layer monitoring scheme monitors the total of N licensed channels in a round robin fashion via a dedicated radio, which we refer to as a the monitoring radio (MR), using the two-state Markov model; the PHY-layer channel monitoring architecture is shown in Figure 3. We use the energy-based PU activity detection method with a detection time of 1 ms [42–44] and 1.16 db energy as a detection threshold to detect either a channel being idle or busy.

**Figure 4:**
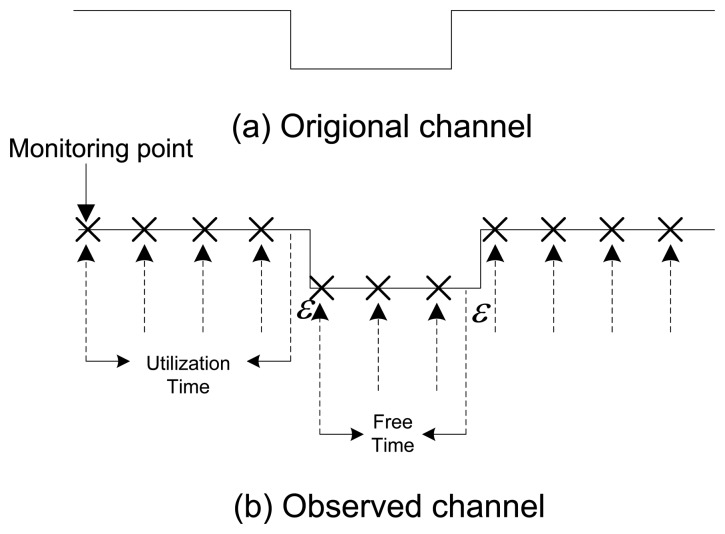
Observing free and utilization times through PU activity monitoring.

The MR monitors all of the licensed channels *i* (*i* = 1, 2,…, *N*) in a round robin fashion [45] and spends a short duration Δ*T*(*e.g.*,Δ*T* = 1) ms over each channel to detect its state. It continues to monitor for the maximum duration of sampling time *T_Sam_* (*i.e.*, 1–5 s) to collect the PU traffic samples from all channels. During the channel monitoring, if a channel *i*is found to be in the ON state (*i.e.*, the detected energy level is greater or equal to 1.16 db for the duration of *T_Sam_*) at time *t*_1_, the MR marks that channel as busy and moves to the next channel. Then, on its next monitoring turn at time *t*_2_, if that channel *i* is still found to be in the ON state, the MR found no change. Therefore, the time *t*_2_ − *t*_1_ is denoted as the utilization time. However, if channel *i* at time *t*_2_ is found to be in the OFF state (*i.e.*, the detected energy level is below 1.16 db for the duration of *T_Sam_*), then time (*t*_2_ − *ϵ*) – *t*_1_ is denoted as the utilization time (where *ϵ* is the detection error). Similarly, if a channel *i* is found to be in the OFF state at time *t*_1_ and then at time *t*_2_ it is again found to be in the same state, then the time *t*_2_ − *t*_1_ is denoted as the free time. However, if channel *i* is found to be in the ON state at time *t*_2_, then the time (*t_2_* − *ϵ*) − *t*_1_ is denoted as the free time. In this scenario, a PU arrival is detected; the counter for the “No. of arrivals” is increased by one. The free and utilization time computation is demonstrated in Figure 4b. Every following encountered utilization or free time lengths will be added in their respective previous values until a different state is found. Finally, Min TOL and Max TOL are estimated through Algorithm 1, every time when the channel switches its state from OFF to ON.

### 3.3. PU Activity-Based Channel Indexing

In this section, we present the channel indexing scheme that facilitates the SUs in selecting the best and most stable channel from the pool of available channels. It only computes the rank or index of the idle channel as follows:

(1) $$\text{Rank}=\frac{\text{TFT}}{\left(\text{TUT}+\text{TNPA}\right)+\text{TFT}}$$

**Figure 5:**
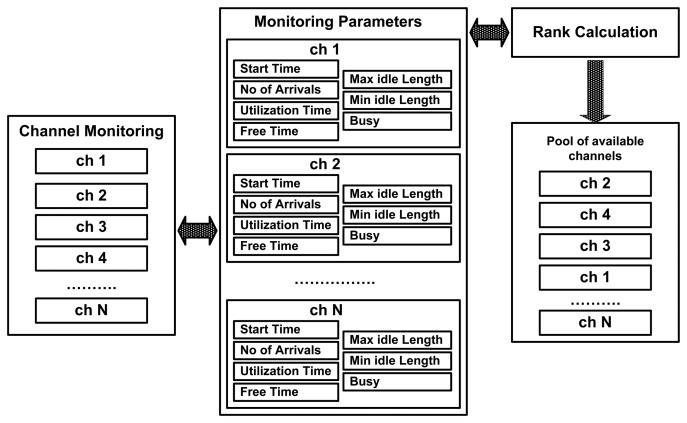
PU activity monitoring and channel indexing architecture.

The rank of each channel is based on three monitored parameters: (1) the total free time (TFT); (2) the total utilization time (TUT); and (3) the total No. of arrivals. The rank gives the confidence value of using any licensed channel *i* for the duration of its estimated Min TOL or, consequently, it refers to the interference value with PU within the estimated Min TOL. Thus, the PAD-MAC protocol selects the optimal channel for enhancing the throughput of the SU based on: (1) the estimated Min TOL; and (2) the rank. The optimal channel selection scheme for transmission of SUs is discussed in Section 3.5. The complete architecture of the activity monitoring of PUs and channel indexing is presented in Figure 5. The pool of available channels *P_ch_* is computed by organizing the idle channels' ranks into descending order. Algorithm 2 demonstrates the method of computing the rank of each available licensed channel.



**Algorithm 2:** Rank computation.
 **Input:**
*Set of Utilization Time, Set of Free Time, Set of No of Arrivals, no of available channels N_a_*, *TMT*, *Set of PUT*, *Set of PFT* and *Set of PNPA* **Output:**
*Set of Rank* **begin**  TUT←0  TFT←0  TNPA ← 0  **for**
*i* ← 1 ***to** N_a_*
**do**   
${\text{Rank}}_{t}^{i}\leftarrow 0$   **if**
*TMT* ==*T_Sam_*
**then**    *TUT^i^* ← *Utilization Time^i^*    *TFT^i^* ← *Free Time^i^*    *TNPA^i^* ← *No of Arrivals^i^*   **else**    *TUT^i^* ← *PUT^i^* + *Utilization Time^i^*    *TFT^i^* ← *PFT^i^* + *Free Time^i^*    *TNPA^i^* ← *PNPA* + *No of Arrivals^i^*   
${\text{Rank}}_{t}^{i}\leftarrow \frac{{\text{TFT}}^{i}}{\left({\text{TUT}}^{i}+{\text{TNPA}}^{i}\right)+{\text{TFT}}^{i}}$


Algorithm 2: The sampling time *T_Sam_* is a global variable, and its value is set according to the total time required for collecting the PHY-layer channel usage patterns. In our case, we set this value to five seconds for the first time; after this, ranks of the available channel can be computed after every second or 500 ms by assigning new monitoring time values to the total monitoring time (TMT) variable.

Moreover, the proposed ranking scheme can be adopted to support the real-time applications over CRNs by selecting a reasonable value of *T_Sam_*. In the case when the TMT is not equal to the sampling time, we compute the ranks of each channel using the past observations of time equal to the sampling time-TMT. The history observations are: (1) previous utilization time (PUT); (2) previous free time (PFT); and (3) previous No. of arrivals (PNPA).

### 3.4. Channel Locking

In CRNs, there are two types of users: (1) PUs, who own the licensed band; and (2) SUs, who opportunistically utilize the PU licensed band. Similarly, CR network performance is affected by two types of interference: (1) PU-to-SU interference; and (2) SU-to-SU interference. PU interference avoidance is the primary objective of CR; therefore, SUs are allowed to use the licensed channels only when their licensees are not using them. However, SU-to-SU interference also severely affects the effective capacity of infrastructure-less CRNs where no central entity exits to manage all of the activities. A few techniques have been proposed to resolve SU-to-SU interference in infrastructure-less CRNs [24,46,47]. However, the PAD-MAC protocol jointly deals with both kinds of interferences as follows:

- PU-to-SU interference avoidance by allowing SUs to exploit the licensed channels only for the duration of their estimated Min TOLs and immediately vacating the channel if the presence of a PU is detected (even if this occurs before the expiration of the estimated Min TOL).
- SU-to-SU interference avoidance by applying the channel-locking scheme during MAC-layer channel sensing and reservation.

MAC-layer sensing is introduced as an additional layer of security for PU-to-SU and SU-to-SU interference avoidance. The channel-locking mechanism aims to eliminate SU-to-SU interference and prevent multiple SUs from accessing a single channel at the same time within the transmission/interference range of each other. In the channel-locking scheme, all SUs who want to transmit data must first participate in contention to acquire access over any particular channel *i*. Each SU node must maintain a variable *cw* (the contention window size), which is initialized with the minimal value initially. Multiple SU nodes can contend for a single channel or for multiple channels depending on which channel is better for them. An SU node first announces contention for its channel of interest and broadcasts a contention message, which includes the channel ID, to all of its one-hop neighbor SU nodes.

All SUs who are also interested in acquiring that particular channel choose a random time value from their *cws* and wait for their corresponding backoff timers to become zero. The counter is deducted by one after each contention slot. The SU node whose backoff timer expires first wins the contention and moves to the next step for MAC-layer channel sensing and reservation. The remaining SU nodes freeze their timers. If any failing SU node is interested in acquiring the next available channel from its list of free channels, it announces the contention over its next channel of interest. In this case, it resumes its frozen timer. In case of a collision, colliding nodes double their contention window sizes. The winning SU proceeds to the MAC-layer channel sensing and reservation phase.

In the MAC-layer channel sensing and reservation phase, the transmitter SU node informs its pair SU node that it wants to communicate on channel *i* by sending the want-to-lock message, which contains channel ID information. If the receiver SU contains this channel *i* in its list of free available channels, it broadcasts the agree-to-lock message. All neighboring SU nodes hearing the want-to-lock or agree-to-lock messages stop their activities over the control channel. After exchanging these control messages, the sender and receiver sense channel *i* for a very short duration *T_s_* to further ensure that there is no PU or SU activity on channel *i*. After sensing *T_s_*, if the sender finds no activity, it sends the lock message containing its estimated *S_MinTol^i^_*; similarly if MAC-layer sensing on the receiver SU node side also is successful (*i.e.*, no activity detected). The receiver SU node first matches its estimated *R_MinTOL^i^_* with the sender estimated *S_MinTOL^i^_* and selects the min of these two values as follow:

(2) $$\mathrm{Min}{\text{TOL}}^{i}=\min\left(S_{\mathrm{Min}{\text{TOL}}^{i}},R_{\mathrm{Min}{\text{TOL}}^{i}}\right)$$

In the response of the lock message, the receiver SU node sends back the locked message along with newly computed *Min TOL^i^* All of the neighboring SU nodes hearing lock and locked messages extract the value of *Min TOL^i^* from the received message, set their network allocation vector (NAV) and also mark channel *i* as unavailable or busy for the duration of *NAV^i^* or until they receive release message. *NAV^i^* is allocated as below:

(3) $${\text{NAV}}^{i}=\mathrm{Min}{\text{TOL}}^{i}$$

In case of PU detection on channel *i*, or expiration of *Min TOL^i^*, or completion of SU transmission, the sender and receiver pair broadcast the release message to inform all of their one-hop neighboring SU nodes so that they unmark/release the marked channel *i* and utilize its remaining idle duration if available; if SU transmission is completed before the expiry of *Min TOL^i^*. On the other hand, if the SU transmission has not been fully completed within the *Min TOL^i^* then the SUs have to follow the contention and channel reservation procedure for the remaining transmissions from scratch.

### 3.5. Predictive and Fast Channel Switching Scheme

Another significant advantage that we attain from our PHY-layer PU activity monitoring and idle slot estimation is enabling the predictive and fast (PF) channel switching/handoff scheme. The prime objective of the PF channel switching scheme is to schedule the transmissions of SUs in such a way as to maximize the SU spectrum occupancy, while minimizing the disruption rate to PUs. For this, each SU first selects a channel from the list of available channels with the highest rank value and remaining idle time *RE_Min TOL_*. The value of $RE_{\mathrm{Min}\text{TOL}}^{i}$ of channel *i* is computed as follows:

(4) $$RE_{\mathrm{Min}\text{TOL}}^{i}=\mathrm{Min}{\text{TOL}}^{i}-T_{\text{wast}}^{i}$$

This means that the consumed idle time (*i.e.*, the time when the channel was already idle while the CR was operating in another channel) $T_{\text{wast}}^{i}$ is subtracted from the estimated idle time Min TOL of any channel *i*. After this, the value of *Min TOL^i^* is updated by assigning the value of $RE_{\mathrm{Min}\;\text{TOL}}^{i}$, the SU computes its channel of interest as follows:

(5) $$Ch_{k}:\arg\underset{i\in N}{\max}\left\{{\text{Rank}}_{i}\times RE_{\mathrm{Min}\text{TOL}}^{i}\right\}$$

In PF channel switching, each communicating SU pair initiates its next channel selection before the expiration of the already in use/reserved channel time in order to avoid any interruption/delay in its ongoing communication. We assume that, on average, channel selection takes time *T_sel_*; therefore, in order to avoid channel selection errors (*i.e.*, channel unavailability), each communicating SU pair computes its new channel selection time *T_sim_* as follows:

(6) $$T_{\text{sim}}=\mathrm{Min}{\text{TOL}}^{i}-2\times T_{sel}$$

**Figure 6:**
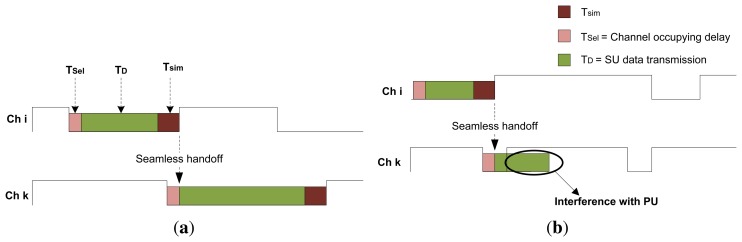
Predictive and fast channel switching scheme. (**a**) Predictive channel switching; (**b**) Predictive channel switching with collision

As the ongoing transmission time reaches point *T_sim_* over the current acquired channel, the communicating SU pair initiates the next/new channel selection to smoothly proceed with its transmission without long delays and/or interruptions. When *T_sim_* is reached, the SU performs both channel selection and data transmission simultaneously and switches to the newly explored channel when *MinTOL^i^* expires. This procedure is presented in Figure 6a,b. Alternatively, in the case of a collision, as shown in Figure 6b, the SU halts its transmission and selects a new channel to resume its communication.

## 4. PU Activity-Aware Distributed MAC for Multi-Channel CRNs

In this section, we discuss the design of the PU activity-aware distributed MAC for a multi-channel CR network. Some necessary design assumptions are listed below:

- We consider a total of *N* licensed channels for data transmissions. The term “channel” refers to a physical channel with a certain amount of bandwidth. We do not consider logical channels (*i.e.*, different coding schemes in code division multiple access (CDMA)). For simplicity, we assume that each spectrum channel has the same bandwidth *B*.
- A common control channel (CCC) is considered to be available for SUs every time. In practice, this can be the unlicensed band [24], which will be used for synchronization and exchanging control information (including contention information).
- PUs have a total of *N* different licensed channels. A single PU can only use one channel at a time. The states of *N* channels at any time *t* and at any location are given by *X*_1_(*t*),*X*_2_(*t*),…,*X_N_*(*t*), where *X_i_*(*t*) is either {*ON*(*busy*)} or {*OFF*(*idle*)}. The channel states are based on the traffic type of PUs, which is modeled using an exponential distribution.
- Each SU node is equipped with two cognitive transceivers. One is for data transmission over the licensed channels, and the second is dedicated to PHY-layer PU activity and traffic analysis.
- When any SU acquires a licensed channel for its transmission, its MR exempts that channel from PHY-layer monitoring for the duration of the reserved time *Min TOL*. This is done by marking the corresponding entry as busy in the monitoring parameters shown in Figure 5.
- The channel ranking algorithm only provides the ranks of available channels at any time *t*. Channels that are busy/unavailable at that time are not considered in channel ranking. Furthermore, if SU transmissions collide with a PU within its estimated idle slot, the rank of the corresponding channel is assigned a value of zero as a penalty.

### 4.1. Protocol Overview

In this section, we first provide a design overview of the proposed PAD-MAC protocol. The estimated idle slot Min TOLs over each licensed channel are referred to as time frames and contain three types of SU operations: (1) contention; (2) MAC-layer sensing and channel reservation; and (3) SU data transmission. These SU operations are depicted in Figure 2. The following packet types are introduced to facilitate these operations:

1. Cont: Before reserving the channel of interest, an SU announces the start of contention for some particular channel *i* by broadcasting the Contmessage if and only if that particular channel is not already marked busy in its local channel base.
2. Want-to-lock: The transmitter SU informs its receiver node and all of its one-hop neighbor SU nodes that a particular channel *i* is going to be reserved. After hearing this message, all of the one-hop neighbor SU nodes stop their activities on the control channel.
3. Agree-to-lock: In response to the want-to-lock message, the receiver SU node broadcasts the agree-to-lock message to inform its transmitter and all of its one-hop neighbor SU nodes if and only if the channel of interest *i* is not already marked as busy in its local channel base and is available in its list of free channels. After hearing this message, all one-hop neighbor SU nodes also stop their activities on the receiver side over the control channel.
4. Lock message: If the transmitter senses no activity on its channel of interest for the duration of its sensing time, it broadcasts the lock message containing the channel ID and idle slot Min TOL information. After receiving this message, all one-hop SU neighbor nodes extract the idle slot information and defer all of their activities by setting their NAV values and marking their corresponding channel IDs as busy in their local channel base.
5. Locked message: If the receiver also does not detect any activity over the channel of interest during the sensing time, then it computes the final conversation/communication time according to Equation (2) and broadcasts the locked message. The lock message contains the channel ID and its conversation time length. All one-hop neighbor SU nodes hearing the lock message also defer their activities and mark this channel as busy in their local channel bases.
6. Release message: Upon the expiration of the reserved time slot or the end of the transmission, the transmitter and receiver broadcast the release message to inform all of their one-hop neighbor SU nodes that channel *i* has been released. All of the SU nodes that hear this message unmark/release the busy channel. The release message is also broadcast in the case of a collision with a PU.

**Figure 7:**
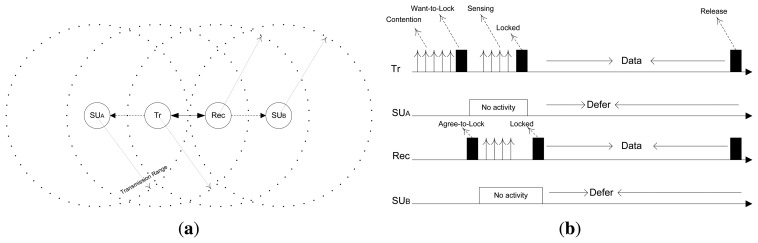
PU activity-aware distributed (PAD)-MAC protocol architecture. (**a**) SU communication ranges; (**b**) PAD-MAC channel reservation and data transmission procedure.

Figure 7a shows that *SU_A_* and *SU_B_* are the two nodes that come under the transmission ranges of *T_r_* and *Rec*, respectively. Therefore, *SU_A_* and *SU_B_* defer their activities as they receive any control message from either the transmitter or receiver. The complete procedure of control message exchange between *T_r_* and *Rec* for channel reservation and data transmission is depicted in Figure 7b. However, all of the aforementioned control messages are exchanged via a CCC. If any SU wants to transmit data, it first reserves the channel after selecting a random backoff time for contention. If it wins the contention, it proceeds to the MAC-layer channel sensing and reservation phase. After successful channel reservation, the transmitter and receiver proceed to data transmission, while their one-hop neighbor SU nodes defer their operations over the reserved channel and wait for the release notification (if they are intending to use the channel in question). If a collision/failure occurs in any of the above control messages, the colliding SUs double their *cws* and proceed to their next channel of interest.

### 4.2. Protocol Design

Licensed channel usage pattern extraction: The traffic usage patterns/features of PUs over all licensed channels are collected through continuous PHY-layer monitoring via a dedicated monitoring radio. The monitoring radio collects the channel features (*i.e.*, utilization time, free time, No. of arrivals, min idle length and max idle length) for the duration of the sampling time. These monitoring parameters are shown in Figure 5. During PHY-layer monitoring, we also estimate the Min TOLs and update their values with respect to any changes that are detected on their respective channels during PHY-layer monitoring. These collected channels' information is used to compute the rank of each individual channel. Rank confers the confidence level or PU collision avoidance value during the estimated *Min TOLs*. Moreover, on the basis of PU channel usage pattern analysis, we can predict the behavior of PU over any particular channel and can predict future idle and busy slots over that channel.

Contention: The PAD-MAC protocol does not require global synchronization. Any new SU node entering the network first listens to the control channel to determine the current activities of its surroundings. A new SU node cannot miss any control packet from its one-hop neighbor SU nodes. If it received any control packet for synchronization, it will stop its activities over the control channel and then defer its activities over the reserved channel after receiving the lock or locked message. Similarly, all of the other SUs also stop their activities over the control and reserved channels and wait for the duration of *NAV^i^* or until they receive the release message from the transmitter or receiver. However, they may proceed to acquire any other channel once the previous synchronization is completed.

If any synchronization occurs in the vicinity of any SU, that node is not allowed to transmit any control packets over the CCC; this avoids unnecessary collisions with previous synchronization. After successful synchronization, the awaiting SU nodes may initiate contention for another channel of their own interest. SU nodes are not allowed to occupy any available licensed channel without announcing and participating in the contention. In the contention period, a media access scheme similar to IEEE 802.11 DCFis adopted (with some necessary modifications). SU nodes can try to reserve available channels, one by one, by exchanging a series of control packets (*i.e.*, Cont, want-to-lock, agree-to-lock, lock and locked). In the case of collisions in any of the control packets, the colliding SU nodes double their contention window size and wait until the backoff timer becomes zero before contending again.

The SU who wins the contention proceeds to the MAC-layer sensing and channel reservation step, while the SU nodes that failed may participate in the contention for other available channels from the same point of their backoff timers after the completion of the previous contention. An SU can only occupy a particular licensed channel maximum for the duration of its estimated *Min TOL*. If it wants to continue its transmission, it may have to follow the whole procedure of channel reservation again for its remaining transmissions. However, an SU node can compute the effective time for data transmission over its channel of interest *i* at time *t* as follows:

(7) $$\mathrm{Min}{\text{TOL}}_{t}^{i}=\mathrm{Min}{\text{TOL}}_{t}^{i}-\left\{T_{cw}+T_{s}+T_{\text{syn}}\right\}$$

where *T_cw_* is the time consumed in contention, *T_s_* is the MAC-layer sensing time to ensure that a channel is idle and *T_syn_* is the time required for synchronization with the pair of SU nodes.

MAC-layer sensing and channel reservation: The transmitter SU node who won the contention will perform the MAC-layer channel sensing and reserve its channel of interest *Ch_k_*. The channel sensing phase contains *T_s_* sensing slots, each of which includes the actual channel sensing (*i.e.*, the detection of channel activity). After successful sensing, the transmitter and receiver nodes proceed. They reserve the channel and negotiate with each other by exchanging lock and locked control packets to become synchronized. the transmitter and receiver nodes can only reserve the channel for communication for the maximum duration of Min TOL that has been estimated over that channel. After the expiration of this reserved time period, they must vacate the channel by broadcasting the release control packet. If the communicating nodes have completed their communication before the expiration of the reserved time period and released the channel, other interested pending SUs can occupy and use the remaining idle length *RIL^i^* if it is sufficiently large for transmitting data packets (*i.e.*, if Equation (9) holds). They can compute the remaining idle length from their *NAV^i^* values and the time when they received the release message as follows:

(8) $${\text{RIL}}^{i}={\text{NAV}}^{i}-\left(T_{\text{Release}}+T_{\text{sel}}\right)$$

(9) $${\text{RIL}}^{i}>T_{\text{pkt}}$$

where *T_Release_* is the time when the SU received the release notification, *T_sel_* is the time required to occupy a licensed channel and *T_pkt_* is the time to transmit a data packet. If the presence of a PU is detected over the communicating channel, the communicating SU nodes must immediately stop their communication, vacate the occupied channel and switch to some other channel for their remaining transmissions. In such a scenario, when switching to a new channel, SUs must follow the complete procedure of acquiring a new channel.

SU transmission: After successful channel reservation and synchronization, the transmitter-receiver pair starts sending and receiving data packets over its channel of interest *Ch_k_* for the maximum duration of *Min TOL^i^*. If the presence of PU is detected over that channel during the packet transmission, the SU immediately stops its communication, vacates that channel and moves to some other suitable channel that is available in its list of free channels.

In the case of normal transmission, an SU pair can only reserve a specific channel for the maximum duration of *Min TOL^i^*. It can send and receive multiple data packets in one reservation slot, but if their complete communication is not possible in one reserved slot, the communicating nodes must occupy another channel of interest before the expiration of the current reserved slot. In such scenarios, our proposed predictive and fast channel switching scheme will be applied. The transmitter/receiver SU pair of nodes broadcast the release control packet in the following three situations:

1. Min TOL*^i^* expires.
2. PU presence is detected on the occupied channel.
3. Communication is completed before the expiration of the reserved time period.

Upon receiving the release notification, all SU nodes that hear the message unmark the channel from their local base and reset their *NAV^i^* values to zero. Algorithm 3 summarizes the complete procedure of the PAD-MAC protocol from the PHY-layer channel monitoring to channel reservation and data transmission. A detailed description of all of the steps and workings of Algorithm 3 is given below.



**Algorithm 3:** PAD-MAC procedure for channel selection and data transmission.
 **Input:**
*Global set of channels N* **begin**  **for**
*i* ← 1 ***to** N*
**do**   /* Compute 
$\mathrm{Min}\;{\text{TOL}}_{t}^{i}$ and 
$\mathrm{Max}\;{\text{TOL}}_{t}^{i}$ using Algorithm 1 */  /* Compute rank of all idle channels using Algorithm 2 */  /* Compute *P_ch_* the pool of available channels*/  /* Compute *Ch_k_* using Equation (5) */  **while**
*P_ch_* ≠ *NULL*
**do**   *P_ch_* ← *P_ch_* − *Ch_k_* /*Exclude channel *Ch_k_* from *P_ch_* */   **if**
*Contention_chk_* == *won*
**then**    /* Exchange control packets want-to-lock and agree-to-lock */    **if**
*Control packet collide* == *TRUE*
**then**     /* Wait for random time, and compute next *Ch_k_* using Equation (5) */      Continue    **if**
*Sensed_Channel_chk_* == *no activity detected*
**then**     /* Exchange control packets lock and locked */     **if**
*Control packet collide* == *TRUE*
**then**       /* Wait for random time, and compute next *Ch_k_* using Equation (5) */      Continue     reserve channel *Ch_k_*    **else**     /* Compute next *Ch_k_* using Equation (5) */     Continue   **else**    /* Compute next *Ch_k_* using Equation (5) */     Continue  **if**
*Reservation of Ch_k_* == *successful*
**then**    /* Compute *Transmission time*^*Ch_k_*^ using Equation (7) */    /* Transmit data packets over channel *Ch_k_* */  **else**   /* Wait random time */   /* Recompute *P_ch_*, and follow channel selection procedure again */  **if**
*Transmission time*^*Ch_k_*^
*reaches to T_Sim_* == *TRUE*
**then**   /* Perform data transmissions and channel selection if required simultaneously */  **if**
*Min TOL*^*Ch_k_*^
*Expires*
**then**   /* Release channel by broadcasting a release message */  **if**
*PU detected on channel Ch_k_*
**then**   /* Stop transmission */   /* Release channel by broadcasting a release message */   /* Assign *Rank_Ch_k__* ← 0 */   /* Recompute *P_ch_*, and follow channel selection procedure from scratch */


Algorithm 3: In the first phase, all of the N licensed channels are monitored via the PHY-layer channel monitoring scheme to estimate the idle slots, and then, based on the monitored parameters, the rank of each idle channel is computed. *P_ch_* is the pool/list of the available channels, which is computed by rearranging the available channels in descending order with respect to their corresponding ranks. After this, an SU computes its channel of interest and announces and participates in contention in an attempt to occupy its channel of interest. If the SU could not successfully occupy its computed channel of interest, it may proceed to compute the next channel of interest from the remaining available channels in the *P_ch_* and continue this process until it occupies some channel or there is no channel left in the *P_ch_*. If an SU is unable to successfully occupy any idle channel, then it must wait for a random amount of time before computing the next *P_ch_*. After successful channel selection, the SU computes its effective transmission time and proceeds to transmit its data packets. If its transmission cannot be completed in one reserved slot, it will start exploring another channel of interest at time *T_sim_*, while simultaneously continuing its transmission on the previously selected channel. It switches to the next channel upon the expiration of the reserved slot. On the expiry of the reserved slot or the PU is present (or is detected), the SU releases the previously occupied channel by broadcasting the release message. In the case of a collision with a PU, the rank of the occupied channel is assigned to zero as a penalty.

## 5. Numerical Results

In this section, we compare the performance of the proposed PAD-MAC protocol with the QC-MAC protocol [29] and listen-before-talk MAC protocol [48]. We measure the performance of the MAC protocols under the three fundamental and important parameters of CRNs: (1) SU throughput; (2) PU-to-SU collision rate; and (3) SU channel switching rate. The results were determined using extensive simulations written in C.

1. Throughput: An important performance metric for CRN MAC protocols is throughput. It is a measure of the amount of successful data packets exchanged between communicating SU nodes pairs in the SU effective transmission time over interval [0, *T*]. Throughput is calculated as below:
  (10) $$Thr_{SU}=\underset{T\to \infty }{\lim}N_{p}\times SU\;\text{effective transmission time in}\left[0,T\right]$$
  where *N_p_* is the number of data packets transmitted per unit time. *N_p_* is based on the bandwidth of the channel; therefore, for simplicity and for fair comparisons, we assume that the capacity of all channels is the same and set as 2 Mbps.
  The SU effective transmission time over interval [0, *T*] is the time in which the SU successfully transmits its data packets without colliding with the PUs. We assume that, on average, a collision takes half of the SU data transmission time in the case of exponential traffic and that the switching time is larger than the transmission time. Thus, the SU effective transmission time is calculated as follows:
  (11) $$\text{SU effective transmission time}=T-\left(\left(\eta \times T_{sw}\right)+(\delta =\frac{T_{D}}{2})+\left(n\times T_{s}\right)\right)$$
  where *T_sw_* and *T_s_* are channel switching and sensing delays, respectively, *η*, *δ* and *n* are the number of switchings, the number of collisions and the number of sensings, respectively.
2. PU-to-SU collision rate: Collisions happen when both PUs and SUs simultaneously transmit on the same channel. The collision rate *ϕ_SU_* between PUs and SUs can be used as a PU protection metric, since the main objective of CR is to avoid harmful interference with PUs and to use the leftover spaces in the licensed bands. The collision rate *ϕ_SU_* is denned as below:
  (12) $$\phi_{SU}=\underset{T\to \infty }{\lim}\frac{\text{Number of SU collided packets in}\left[0,T\right]}{T}$$
3. SU channel switching rate: Each incidence of channel switching causes some delay in the SU transmission, thereby causing wastage of the SU effective transmission time. Frequent channel switching decreases the capacity and makes the network management more difficult [49]. Thus, a good metric for the spectrum control in CRNs is the channel switching rate *θ_SU_*. This is a measure of the number of channel switchings in interval [0, *T*]. Minimizing the channel switching rate *θ_SU_* also decreases the probability of collisions with PUs. *θ_SU_* is computed as follows:
  (13) $$\theta_{SU}=\underset{T\to \infty }{\lim}\frac{\text{Number of SU channel switching in}\left[0,T\right]}{T}$$

### 5.1. Experimental Setup for Channel Monitoring and Ranking

In this section, we discuss the environmental setup for the simulation of our proposed PHY-layer channel monitoring scheme. We use an energy-based PU signal detection scheme with a 1-ms detection time and 1.16 db as the detection threshold [42]. If the detected energy value is below this threshold, we assume that the channel is idle; if the detected energy is equal to or above the detection threshold, then we assume that the channel is busy. To demonstrate our PHY-layer channel monitoring scheme for PU traffic usage pattern analysis and rank calculation, we used five licensed channels. PU traffic is exponentially modeled using alternating ON-OFF states [41]. The lengths of the ON-OFF states are also exponentially distributed with idle rate λ*_TOFF_* and busy rate λ*_TON_*, respectively, which are independently chosen for each individual channel. The sampling time for the collection of the PU channel usage pattern is set as 5 s; an SU will have to wait for the sampling time *T_Sam_* to collect the samples; however, the following time, an SU can compute the ranks of available channels after every second. This can be done by using the history of the previous 4 s.

### 5.2. Experimental Setup for SU Channel Selection and Data Transmission

In this section, we discuss the simulation setting for the channel selection and data transmission of SUs in the proposed PAD-MAC protocol. Simulation parameters and their corresponding vales are presented in Table 2. SU nodes are randomly placed in a 1000 × 1000 m area of two dimensions. An SU can directly communicate with its respective destination SU node within a transmission range of 150 m. For channel selection and data transmission, we used a total of 10 heterogeneous licensed channels (*i* = 1, 2,‥, 10), over which PU activities are exponentially distributed with different values of mean idle $\frac{1}{\lambda_{T_{\text{OFF}}}^{i}}$ and mean busy $\frac{1}{\lambda_{T_{ON}}^{i}}$ periods for each individual channel i. $\lambda_{T_{\text{OFF}}}^{i}$ and $\lambda_{T_{ON}}^{i}$ denote the busy and idle rates of the exponential distribution for each individual channel i. Alternatively, the values of $\lambda_{T_{\text{OFF}}}^{i}$ and $\lambda_{T_{ON}}^{i}$ are Presented in Table 3. For simplicity and fair analysis, the capacity of each channel is assumed to be equal and set to 2 Mbps.

We tested the performance under different channel conditions (listed above) by varying the total number of channels to be used: (1) three channels (*i* = 1,2,3); (2) five channels (*i* = 1,2,3,4,5); (3) seven channels (*i* = 1, 2, 3, ‥‥, 7); and (4) ten channels (*i* = 1, 2, 3,4, ‥‥, 10). *T_s_* is the average time spent in channel selection and reservation for SU packet transmission. Therefore, for a fast/seamless channel handoff, the communicating SUs start exploring their next channels at time *T_sim_* in order to obtain smooth communication. Hence, from the point *T_sim_* onward, SU nodes perform data transmission and channel selection simultaneously. If any SU quits its reserved channel due to collision with a PU, then the SU must wait for 1 s for PHY-layer channel monitoring; 5 ms is used for channel ranking, next channel selection, synchronization and switching. We modeled error-free channels and assumed that packet loss only occurs due to PU-to-SU collisions. We ran simulations for 250 s to measure the performance values. To observe the average behavior, simulations were repeated under the same condition 10 times. The PAD-MAC protocol is evaluated against the QC-MAC and listen-before-talk MAC schemes under a multi-channel CR network environment. The performance is evaluated using file transfer data traffic with a constant packet length of 1500 bytes. QC-MAC computes the success probabilities of each packet transmission over a list of available channels and selects a channel with a higher probability of success. Probability values are computed using the mean idle, mean busy time of traffic parameters *T_ON_*; *T_OFF_*, sensing time and packet transmission time as follows:

(14) $$P_{j}^{i}=\frac{\beta_{i}}{\alpha_{i}+\beta_{i}}F_{i}\left(t_{AS_{j}}+t_{f}\right)$$

where $\frac{1}{\alpha_{i}}$ and $\frac{1}{\beta_{i}}$ are the mean idle and busy periods of channel i, respectively. *t_As_j__*, is an arbitrary sensing period selected by user *j*. *t_f_* is the transmission time of the current frame, and *F_k_*(*x*) is the cumulative distribution function.

Thus, a total of 2 ms is reserved for channel selection (*i.e.*, computing success probabilities, sensing and synchronization), and 1 ms of additional time will be added in the case of switching to a new channel. A random time is picked from the waiting window of size [0, 8] ms when no idle channel is available for selection. In the case of a collision with a PU, no additional time penalty is awarded in the QC-MAC, because this scheme senses the channel every time before sending each individual data packet. The collision probabilities (corresponding to QC-MAC success probability INLINE) over any channel *i* are given in Table 4. The same collision probabilities are used for PAD-MAC based on its rank index; however, the collision probabilities for the listen-before-talk MAC are randomly selected for every channel. Similarly, the listen-before-talk MAC scheme senses the licensed channel each time before sending the data packet. It only differs with the QC-MAC in that it does not compute the transmission success probabilities over all available channels. It only senses the channels in a descending order and picks up the first encountered available/idle channel. One millisecond is used to sense a single channel to determine whether it is idle or busy; if the sensed channel is busy, it moves to the next channel and continues searching until either an idle channel is found or all channels have been analyzed. If all of the channels are found to be busy or if a collision with a PU occurs, it waits for a random time period selected from the time window of size [0, 8] ms, similar to the QC approach. Two milliseconds is used for channel selection, synchronization and switching to the new channel.

## 6. Results

### 6.1. Channel Monitoring and Ranking

**Figure 8:**
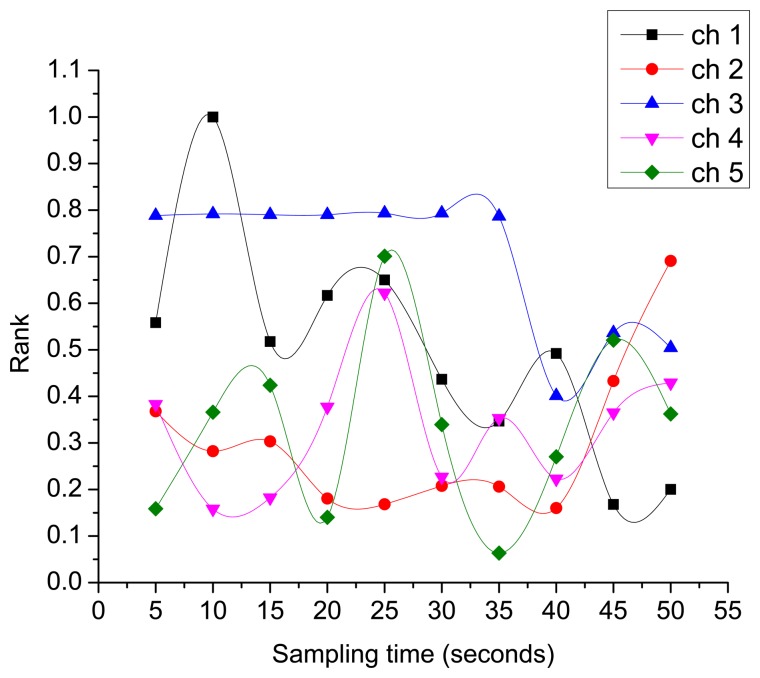
Dynamic behavior of channel ranks.

Figure 8, presents the dynamic behavior of PU activity-aware channel ranks. This figure shows that as the activities of the PUs changes over time, there is a pertinent change in their corresponding ranks. Thus, selecting a channel based on its rank value provides the best and most stable channel for SU transmissions, which significantly enhances the throughput of CRNs.

**Figure 9:**
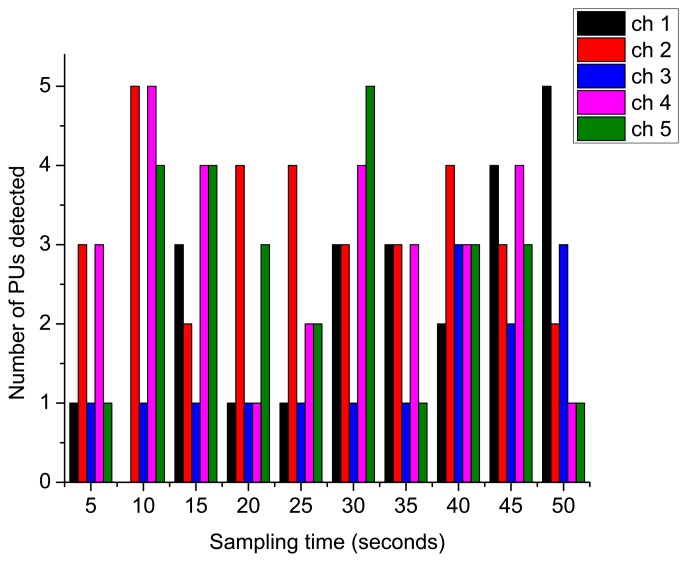
Detected PU arrivals.

Figure 9 shows the number of PU arrivals detected during the monitoring of the PHY-layer PU activity over the licensed channels. The number of detected arrivals is grouped together over the period of the sampling time.

**Figure 10:**
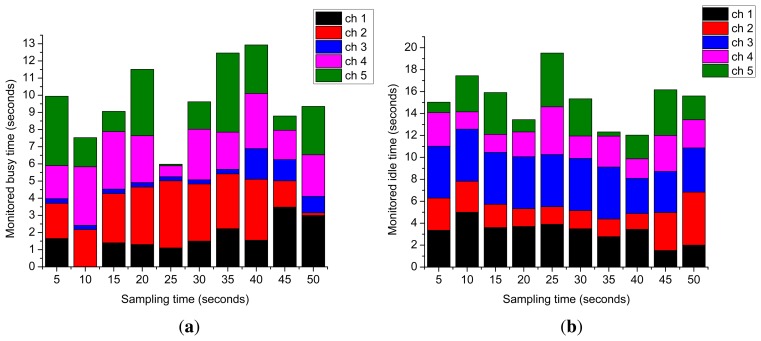
Monitored idle and busy time. (**a**) Detected total busy time; (**b**) detected total idle time.

Figure 10a,b presents the total busy and idle times, respectively, which are observed for each individual monitored licensed channel during PHY-layer monitoring over a total simulation time of 50 s. Each channel that is determined to be busy or idle is grouped together over a sampling time of 5 s. These two figures reveal the fact that different licensed channels have different usage and leftover times, and they vary over time with PU activity in their respective channels. Therefore, this fact can play a significant role in enhancing the throughput of opportunistic access CRNs.

**Figure 11:**
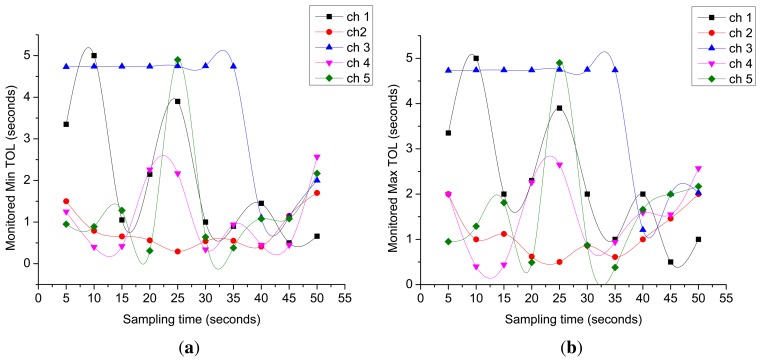
Monitored Min TOLs and Max TOLs. (**a**) Monitored Min TOLs; (**b**) monitored Max TOLs.

Figure 11a,b presents the estimated time lengths of Min TOLs and Max TOLs estimated for each monitored licensed channel over a total simulation time of 50 s. We estimate these two values every time we detect a change in their respective channels. *Min TOL^i^* refers to the expected idle time period that is available in channel *i*; this is the actual time that an SU can utilize for its own data transmission. These two figures demonstrate that Min TOLs and Max TOLs can also vary over time the activity of the PU (*i.e.*, idle and busy times) changes over their respective licensed channels.

Our calculated rank for each channel basically refers to the confidence value of using that licensed channel for the minimum duration of its estimated Min TOL time. Thus, a higher rank value means that the estimated idol slot will be less affected by its PU and has a higher chance of successful communication in that idle slot. Selecting a stable channel not only minimizes the number of channel handoffs, but also enhances the SU throughput.

**Figure 12:**
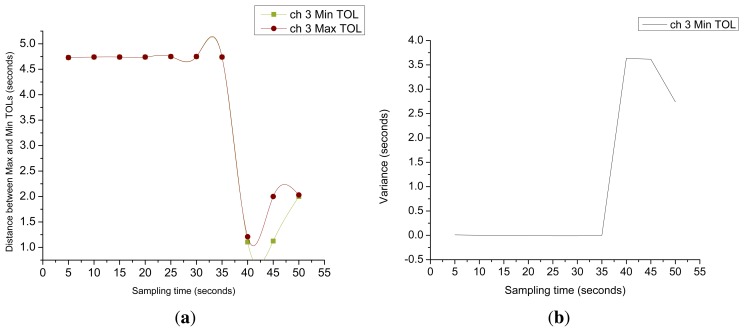
Distance and variance of Ch 3. (**a**) Distance between Ch 3 estimated Min TOL and Max TOL; (**b**) variance in Ch 3 estimated Min TOL.

Figure 12a shows the distance between measured Min TOL and Max TOL on Channel 3 over a set of observed point *p* = {1,……, *M*}. The distance *Dist^i^* between the two measured entities is computed as follows:

(15) $${\text{Dist}}^{i}=\sum_{p=1}^{M}\left(\mathrm{Max}{\text{TOL}}_{p}-\mathrm{Min}{\text{TOL}}_{p}\right)$$

if *Dist^i^* for any given channel *i* is approximately equal to zero over a period of time then the channel having the same idle lengths (*i.e.*, same length of *Min TOL* and same length of *Max TOL*). Therefore, such a channel is considered to be more stable than the channel having different idle lengths. The standard deviation INLINE of Min TOLs of any given channel i over a set of monitored points *p* = {1,……, *M*} is computed as follows:

(16) $$\sigma_{\mathrm{Min}\text{TOL}}^{i}=\sqrt{\frac{1}{M}\sum_{p=1}^{M}{\left({\mathrm{Min}\text{TOL}}_{p}-\mu \right)}^{2}}$$

where *μ* is the mean value of measured Min TOLs, which is computed as below:

(17) $$\mu =\frac{1}{M}\sum_{k=1}^{M}\mathrm{Min}TOL_{k}$$

If the *Dist^i^* computed in Equation (15) and $\sigma_{\mathrm{Min}\;\text{TOL}}^{i}$ computed in Equation (16) are approximately equals to zero for any given channel *i*, we can conclude that the behavior of the PU over that channel is periodic.

Figure 12b demonstrates the behavior of $\sigma_{\mathrm{Min}\;\text{TOL}}^{3}$ and we can observe that its value remains approximately zero in the interval between 5 and 35 s. Therefore, we can conclude that the PU behavior over Channel 3 remains almost periodic within that interval. If such a channel were found, then we can make exact predictions about the future idle and busy slots to better exploit the channel for SU transmissions and to avoid PU-to-SU interference. For this case, the prediction about the start of the ON time or the point where the SU and PU transmissions can collide with each other can be calculated as follows:

(18) $$T_{PU}=m\times \mathrm{Min}\text{TOL}+T_{ON}$$

where *m* = {1, 3, 5, … } is the set of odd numbers and *Ton* is the channel busy time. We assume that *Ton* appears before every idle time Min TOL. Similarly, the start of a future idle time is computed as below:

(19) $$T_{\text{Idle}}=\left(n\times \mathrm{Min}\text{TOL}+T_{ON}\right)-1$$

where *n* = {2, 4, 6,… } is the set of even numbers.

### 6.2. SU Channel Selection and Data Transmission

**Figure 13:**
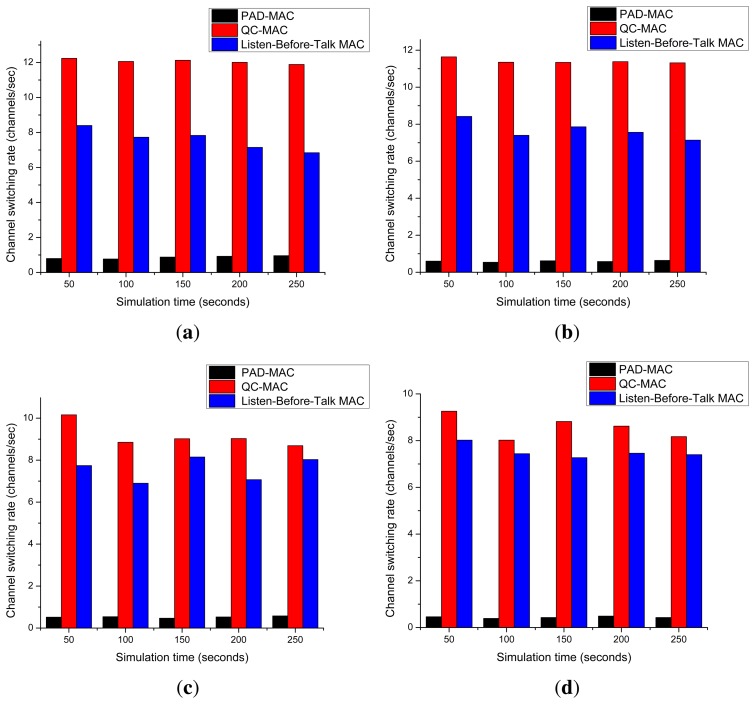
Channel switching rate. (**a**) Three channels; (**b**) five channels; (**c**) seven channels; (**d**) ten channels.

Figure 13 presents the channel switching rate for the three, five, seven, and 10 channel scenarios. It shows that the PAD-MAC scheme outperforms the QC-MAC and listen-before-talk MAC schemes. The channel switching rate of QC-MAC is very high; for every packet transmission, it computes the success probabilities for all available channels and selects the channel with the highest probability of success. However, under the dynamic channel condition, the probabilities vary almost every time they are computed.

The channel switching rate of the listen-before-talk scheme is comparatively lower than the QC-MAC, because it senses the licensed channels in any order (*i.e.*, descending or ascending), picks the first found idle channel and transmits its packet. For the next packet transmission, it again senses the same channel; if that channel is still found to be idle, it sends the next packet over the same channel again, continuing to hold the occupied channel until it becomes busy or a collision occurs over that channel. The channel switching rate of the PAD-MAC scheme is much lower than either of the other two schemes, because it occupies the complete idle slot (*i.e.*, the estimated Min TOL) and transmits as many packets as it can within that idle slot.

**Figure 14:**
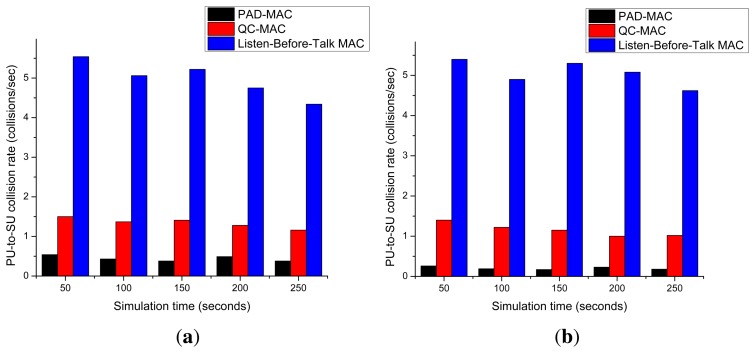

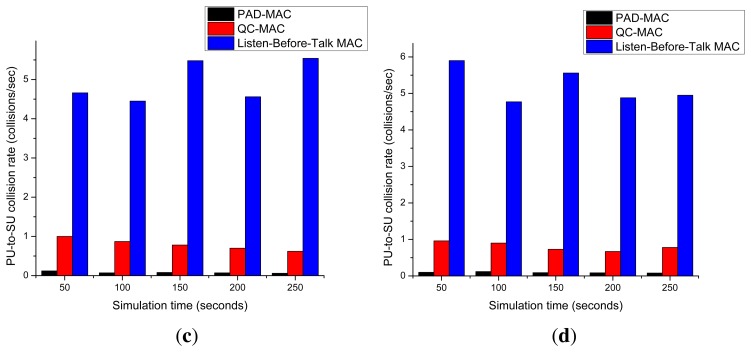
PU collision rate. (**a**) Three channels; (**b**) five channels; (**c**) seven channels; (**d**) ten channels.

Figure 14 shows the collision rate measured for the three, five, seven and 10 licensed channel scenarios. This figure also provides evidence of the better performance of the PAD-MAC during collisions. The collision rate of the listen-before-talk MAC protocol is much higher than the other two MAC schemes, because unlike QC-MAC and PAD-MAC, it selects the channel without taking any precautionary measurements. The reason for fewer collisions in the proposed PAD-MAC scheme is that we estimate the idle slot through PHY-layer monitoring and select the channel based on its rank. As the number of licensed channels increases, the collision rate of QC-MAC decreases; this is because it has a wider range for channel selection and picks a good channel with a higher probability for success. PAD-MAC has better performance while experiencing collisions compared to the QC-MAC, because in QC-MAC, collisions occur per packet transmission; in PAD-MAC, the collisions occur per idle slot.

**Figure 15:**
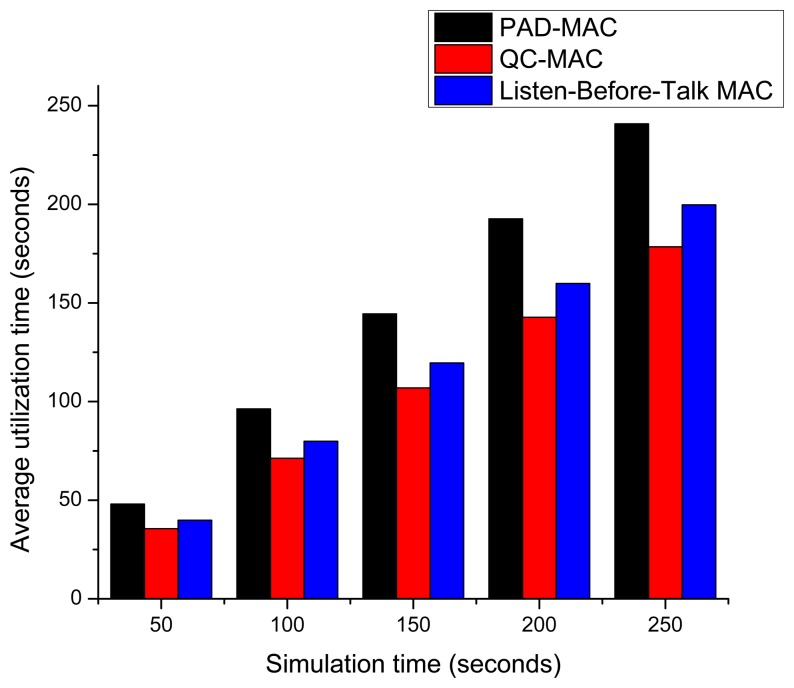
Average SU effective transmission time.

Figure 15 presents the average utilization or average effective transmission time. The PAD-MAC effective transmission time is higher than the other two schemes because of its low collision and channel switching rates. Moreover, PAD-MAC exploits the predictive and fast channel scheme; the other two MAC schemes spent much of their effective time just in selecting and switching to new channels.

**Figure 16:**
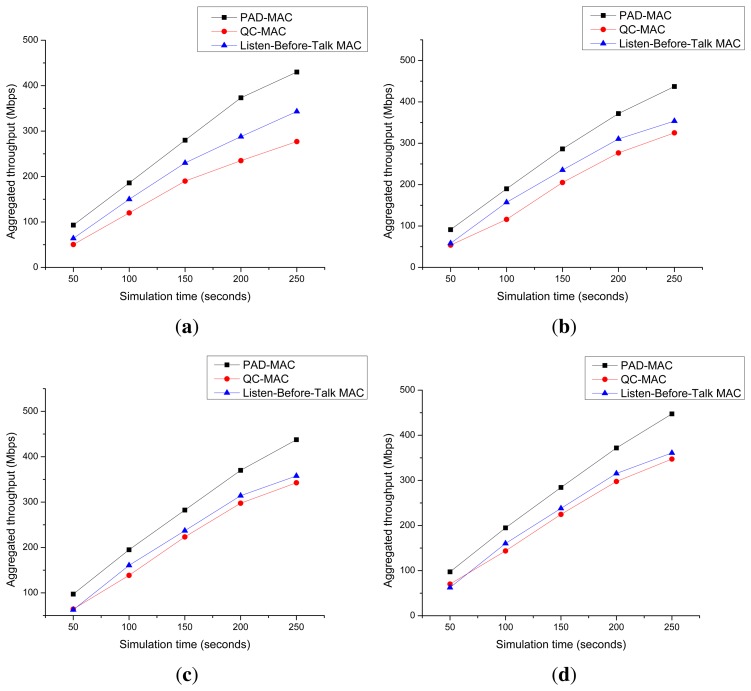
Aggregated throughput. (**a**) Three channels; (**b**) five channels; (**c**) seven channel; (**d**) ten channel.

Figure 16 presents the aggregated throughputs for three, five, seven and 10 licensed channel scenarios. It is clear from the figure that the aggregated throughput of our proposed PAD-MAC is higher than the listen-before-talk and QC-MAC protocols.

## 7. Conclusions and Future Work

In this paper, we have proposed a MAC protocol (PAD-MAC) that utilizes multiple licensed channels to improve CRN throughput and overall spectrum utilization. We take several considerations to make the CR more practical, including monitoring the channel activities of primary users and estimating idle slots based on previous channel observations. Similarly, based on the past observations of the channels, we computed the rank of each individual available channel, which assesses the transmission success of SUs within an estimated idle slot over any particular licensed channel. Thus, each SU selects the channel based on the estimated idle slot length and rank value to optimize its throughput. Our proposed PAD-MAC protocol also coordinates the contention, MAC-layer channel sensing, synchronization and negotiation for spectrum usage among multiple SUs. It controls the activity of SUs over the licensed channels to avoid harmful interference with PUs by allowing the SUs to exploit the licensed channels only for the duration of the estimated idle time. Moreover, communicating SU nodes use the predictive and fast channel switching scheme to maximize their effective transmission time and capacity. Simulation results show the significant improvement in the throughput of SUs for various system configurations. As future work, we would like to extend our study by focusing on the PU activities specific to their practical applications (*i.e.*, real traffic patterns) and considering the fairness among multiple SUs.

## Figures and Tables

**Table 1:** List of symbols.

N	Number of licensed channels
B	Bandwidth of channel
*X_i_*(*t*)	State of channel i at time t
MR	Monitoring radio
*T_Sam_*	Total sampling time to collect the channel usage patterns
*ϵ*	Detection error
Min TOL	Minimum transmission opportunity length
Max TOL	Maximum transmission opportunity length
*P_ch_*	Pool of available channels
cw	Contention window size
*T_s_*	MAC-layer channel sensing time
*S_MinTOL^i^_*	Lransmitter SU measured Min TOL over channel i
*R_MinTOL^i^_*	Receiver SU measured Min TOL over channel i
*MinTOL^i^*	Min of *S_MinTOL^i^_* and *RM_inTOL^i^_*
*NAV^i^*	Network Allocation Vector corresponding to channel i
(inline)	Remaining Min TOL over channel i
*T_cw_*	Time consumed in contention
*Ch_k_*	Channel of interest
(inline)	Time wasted when channel i is idle while the SU is operating on some other channel
*T_sel_*	Average time used for channel selection
*T_sim_*	Time when SU performs transmission and next channel selection simultaneously
*T_sym_*	Time required for synchronization
*T_pkt_*	Packet transmission time
*N_p_*	Number of packets transmitted in unit time
*η*	Number of channel switching
*δ*	Number of collisions
*ϕ_SU_*	Collision rate
*θ_SU_*	Channel switching rate

**Table 2:** Simulation parameters.

Simulation total time	250 s
Simulation area	1000 × 1000 m
SU transmission range	150 m
Number of licensed channels	10 channels
Channel capacity	2 Mbps
Number of PUs	10 (each licensed channel corresponds to its single PU)
Number of SUs	25
Packet length	1500 bytes
PU detection time	1 ms
Detection threshold value	1.16 db
Sampling time for the collection of PU channel usage patterns	5 s
Time required for channel ranking, selection and switching	5 ms
Waiting window size in the case of no idle channel being available	[0, 8] ms

**Table 3:** Channel usage pattern.

**Channel**	λ*_T_OFF__*	λ*_T_ON__*	**Channel**	λ*_T_OFF__*	λ*_T_ON__*
Ch1	0.215	0.4	Ch6	0.21	0.31
Ch2	0.354	0.4	Ch7	0.65	0.4
Ch3	0.11	0.982	Ch8	0.26	0.31
Ch4	0.251	0.45	Ch9	0.42	0.24
Ch5	0.51	0.14	Ch10	0.312	0.217

**Table 4:** PU interference probability index table.

$x=P_{i}^{k}$	***P****_r_* **(*PU Inter ference*)**
*x* ≤ 0.4	0.6
0.4 < *x* ≤ 0.8	0.3
0.8 < *x* ≤ 1.0	0.1
